# Deciphering the Association Between Homeostatic Model Assessment of Insulin Resistance and Chronic Diarrhea and the Mediating Role of Lymphocyte-to-Monocyte Ratio

**DOI:** 10.5152/tjg.2026.25247

**Published:** 2026-01-26

**Authors:** Yuqun Chen, Ye Xiao, Zongtao Chen

**Affiliations:** Health Management Center, First Affiliated Hospital, Army Medical University, Chongqing, China

**Keywords:** Chronic diarrhea, HOMA-IR, insulin resistance, lymphocyte-to-monocyte ratio, National Health and Nutrition Examination Survey

## Abstract

**Background/Aims:**

Metabolic disorders and insulin resistance (IR) may elevate the risk of chronic diarrhea (CrD), yet the association between the homeostatic model assessment of IR (HOMA-IR) and CrD remains unclear. This study aims to examine the HOMA-IR-CrD relationship and investigate the mediating role of lymphocyte-to-monocyte ratio (LMR).

**Materials and Methods:**

A total of4984 participants from the National Health and Nutrition Examination Survey database were included. The weighted logistic regression analysis was performed to assess the linkage between HOMA-IR and CrD. Through subgroup analyses, differences in various populations were evaluated. Moreover, mediation analysis was conducted to assess the mediating role of LMR.

**Results:**

After adjusting for all covariates, HOMA-IR was positively linked with CrD (OR = 1.03, 95% CI: 1.00-1.05). The risk of CrD in the fourth quartile array of HOMA-IR was 1.68 times higher than that in the first quartile array (95% CI: 1.03-2.74). The subgroup analysis revealed a positive correlation between HOMA-IR levels and CrD in females (OR = 1.04, 95% CI: 1.00-1.08), people with a university and above educational background (OR = 1.06, 95% CI: 1.01-1.12), and the current smoking population (OR = 1.07, 95% CI: 1.03-1.12). Furthermore, LMR partially mediated the relationship between HOMA-IR and CrD, with a mediation ratio of 3.76%.

**Conclusion:**

Elevated HOMA-IR is significantly linked with the risk of CrD, especially in women, people with a university or higher background, and current smokers. The LRM assumes a partial mediating role in that process. Interventions targeting IR may contribute to the management and prevention of CrD.

**Main Points**

- Homeostatic model assessment of insulin resistance (HOMA-IR) is positively correlated with chronic diarrhea (CrD), especially in females, those with a university or above education background, and current smokers.
- Lymphocyte-to-monocyte ratio plays a partial mediating role in the association between HOMA-IR and CrD.

## Introduction

Chronic diarrhea (CrD) is a common gastrointestinal symptom with symptoms lasting for more than 4 weeks,^1^ posing a daunting impact on patients’ quality of life.^2^ In recent years, the potential association between metabolic disorders and CrD has garnered increasing attention.^3^^,^^4^ Individuals with diabetes have been found to possess a higher risk of developing CrD compared to non-diabetic individuals.^5^ Insulin resistance (IR), as one of the core mechanisms underlying type 2 diabetes, has its marker triglyceride–glucose (TyG) index positively correlated with CrD.^6^ However, the direct relationship between the application of the homeostasis model assessment of IR (HOMA-IR) and CrD is not yet clear.

Insulin resistance may affect gastrointestinal function through multiple pathways, among which inflammatory and immune mechanisms are increasingly being emphasized. HOMA-IR is proven to be linked with systemic inflammation, and chronic inflammation is one of the key factors leading to intestinal dysfunction.^7^ The lymphocyte-to-monocyte ratio (LMR) is an easily accessible biomarker for systemic inflammation and immune balance. Its inverse correlation with ulcerative colitis disease has been discovered.^8^ The LMR may play an important role in intestinal health. Based on this, LMR may be an important mediating indicator linking IR and CrD. However, there is currently no systematic research exploring whether LMR plays a mediating role in the association between HOMA-IR and CrD.

Given this, the National Health and Nutrition Examination Survey (NHANES) database in the United States was utilized to explore the linkage between HOMA-IR and CrD and to further analyze the mediating role of LMR. This work aims to provide new scientific evidence from the perspectives of metabolism and immune inflammation for the prevention and management of CrD.

## Materials and Methods

### Study Population

**Figure 1.**
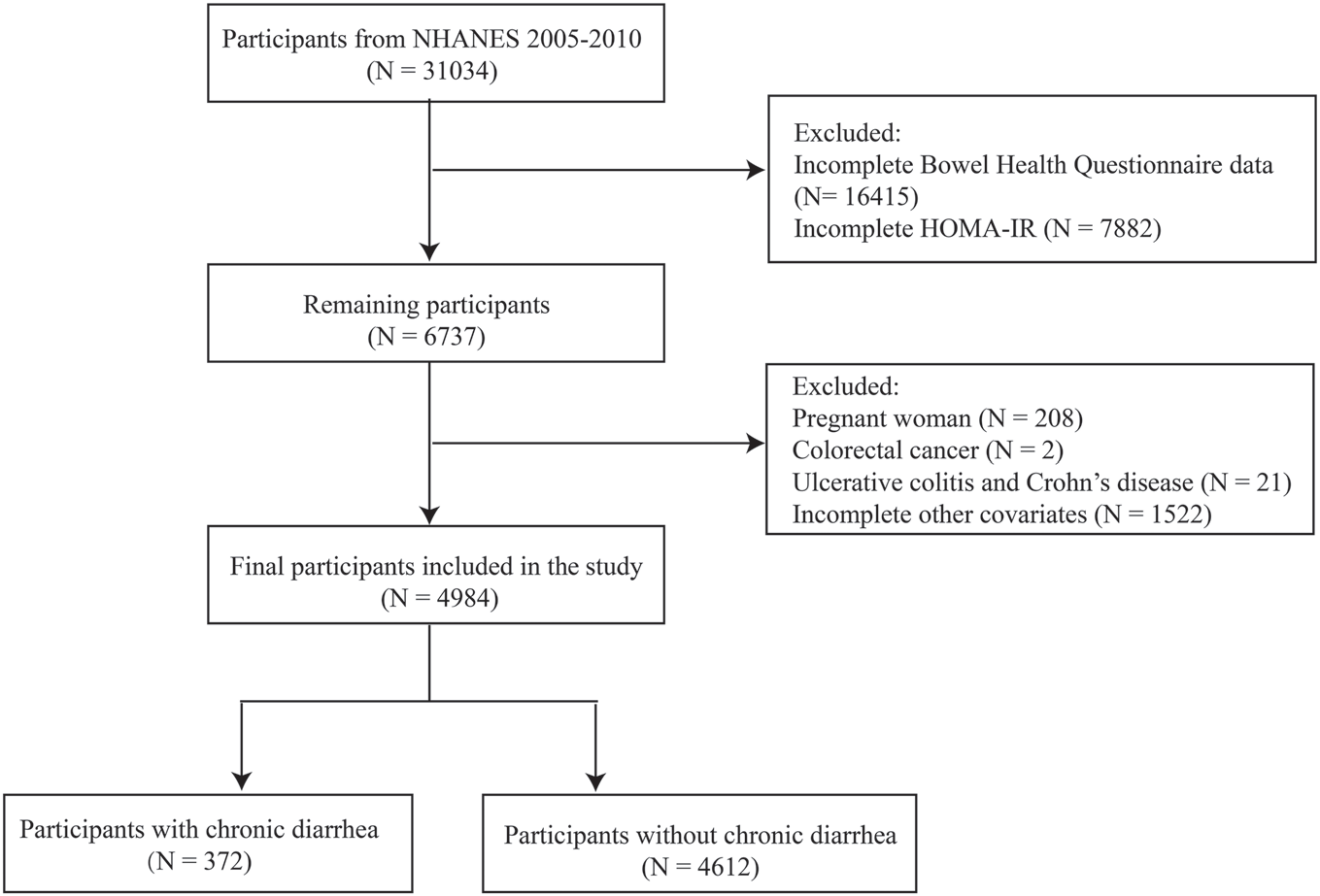
NHANES 2005–2010 patient cohort and analytic samples.

The data for this study was extracted from the NHANES database from 2005 to 2010 (https://www.cdc.gov/nchs/nhanes/index.html). The NHANES is a comprehensive study conducted by the Centers for Disease Control and Prevention and the National Center for Health Statistics, with a complex multi-stage, stratified, and probabilistic sampling design to ensure data representativeness. Data were collected through home interviews, standardized physical examinations at the mobile examination center (MEC), and laboratory testing. After excluding participants with incomplete gut health questionnaire data (n = 16 415), participants without HOMA-IR (n = 7882), pregnant women (n = 208), colorectal cancer patients (n = 2), patients with ulcerative colitis and Crohn’s disease (n = 21), and participants without other covariates (n = 1522), a total of 4984 participants were enrolled in the study (Figure 1).

### Independent Variables, Dependent Variables, and Mediating Variables

The independent variable in this study was the HOMA-IR index. The calculation formula is HOMA-IR = fasting blood glucose (mmol/L) × fasting insulin (μU/mL)/22.5.^9^

The dependent variable CrD was defined based on the gut health questionnaire survey conducted in MEC interviews (Supplementary Material). The staff at the MEC provided the subjects with a card with a colored image and a description of the Bristol Stool Form Scale (BSFS) types (1-7). Participants were diagnosed with CrD only when they reported that their typical or most common stool type was BSFS 6 (fluffy edges, pulpy) or 7 (watery, no solid mass).^10^

The mediator variable was LMR, calculated as the ratio of lymphocyte count to monocyte count.^11^ These 2 counts were both from automated whole blood cell count performed on blood samples collected during MEC physical examination.

### Covariates

The covariates of this study included age, gender, race, education level, marital status, poverty income ratio (PIR), a body shape index (ABSI), smoking status, alcohol consumption, depression, vigorous physical activity, total sugar intake, dietary fiber intake, total fat intake, caffeine intake, water intake, and LMR. According to PIR, participants were clustered into 3 groups: low-income (≤1.3), middle-income (1.3-3.5), and high-income (>3.5).^12^ The ABSI was calculated using the formula 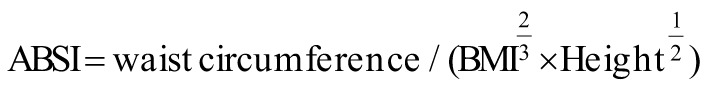.^13^ Participants were categorized into groups of never smoking, previous smoking, and current smoking according to their smoking habits. Never smoking was defined as not having smoked up to 100 cigarettes in their lifetime. People who had smoked up to 100 cigarettes in the past but currently do not smoke were classified into the previous smoking group. Current smokers were defined as individuals who currently smoke every day or several days.^14^ Participants who consumed at least 12 standard cups of alcoholic beverages per year were defined as those with drinking habits.^15^ Depression was evaluated through the Patient Health Questionnaire-9 (PHQ-9), with a PHQ-9 score ≥ 10 indicating the presence of depression.^16^ The definition of intense physical activity varied depending on the survey cycle. In the 2005-2006 period, it referred to any activity lasting for over 10 minutes and causing substantial sweating or a great increase in heart rate and breathing, performed at least once within the past 30 days. In the 2007-2010 cycle, it referred to situations where there was a great increase in breathing or heart rate during work or leisure activities.^17^

### Statistical Analysis

The data processing and statistical analysis in this study were conducted using R software version 4.4.2 (R Foundation for Statistical Computing; Vienna, Austria). The “gtsummary” package (https://cran.r-project.org/web/packages/gtsummary/refman/gtsummary.html) was employed to create a baseline table and group respondents based on their overall population characteristics regarding their CrD status. Categorical variables are represented by sample size and weight-adjusted proportion, while continuous variables are represented by weight-adjusted mean and standard deviation. A weighted logistic regression model was established by using the “survey” package (https://cran.r-project.org/web/packages/survey/refman/survey.html) to probe into the association between HOMA-IR and its quartiles with CrD. Data were weighted (WTSAF2YR), and the confounding factors were gradually adjusted in 2 different models. Model I had adjustments in age, gender, race, and educational level, while Model II had adjustments in all relevant covariates. In the model adjusted for all confounding variables, the “rms” package (https://cran.r-project.org/web/packages/rms/refman/rms.html) was applied in the restricted cubic spline analysis (knots = 3) to illuminate the nonlinear relationship between HOMA-IR and CrD. In addition, a stratified multivariate logistic regression model was employed for subgroup analysis. Finally, the “mediation” package (https://cran.r-project.org/web/packages/mediation/vignettes/mediation.pdf) was employed to investigate the mediating role of LMR in the relationship between HOMA-IR and CrD. Non-parametric Bootstrap method was applied, and 1000 times repetition was ensured to estimate indirect effects and a 95% CI. *P *< .05 indicates significant differences. Since the data is publicly available, ethics committee approval and informed consent was not required.

## Results

### Baseline Characteristics

This study included 4984 participants with an average age of 46.99 ± 16.28 years. Participants were grouped according to the presence of CrD; 372 individuals (6.5%) had CrD. The proportion of CrD patients who currently smoke (29.0% vs. 21.0%, *P *= .024) and suffer from depression (14.0% vs. 5.7%, *P *< .001) was significantly higher than that of non-CrD participants. Moreover, the HOMA-IR of patients with CrD was significantly higher than that of non-CrD patients (3.99 vs. 3.19, *P *= .003) (Table 1).

### Association Between Homeostatic Model Assessment of Insulin Resistance and Chronic Diarrhea

The association analysis between HOMA-IR and CrD was carried out (Table 2). In the model adjusted for all confounding factors, HOMA-IR was significantly positively linked with CrD (OR = 1.03, 95% CI: 1.00-1.05, *P *= .022). When HOMA-IR was used as the categorical variable, all 3 models exhibited a great increase in the risk of CrD among participants in the fourth quartile array compared to the first quartile array. After adjusting for all confounding factors, the risk of CrD in the fourth quartile array was 1.68 times higher than that in the first quartile array (95% CI: 1.03-2.74, *P *= .040).

**Figure 2.**
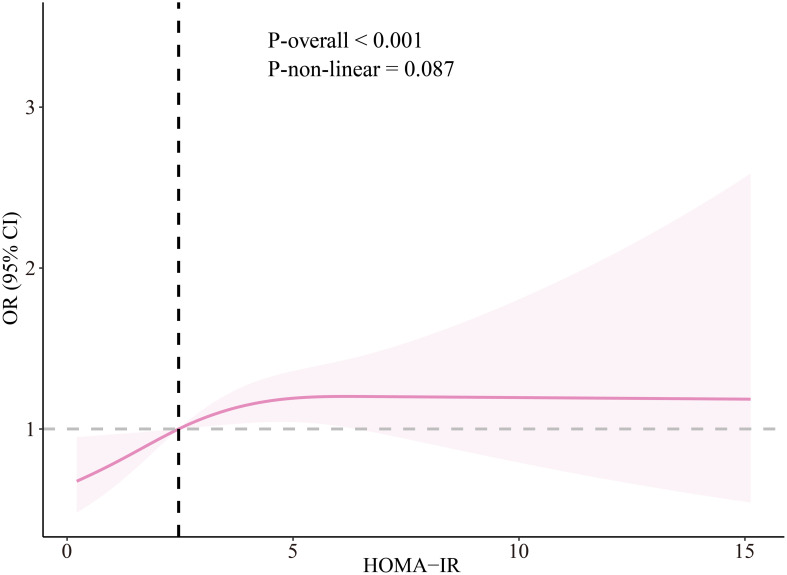
The odds ratio of HOMA-IR and CrD adjusted by covariates, NHANES 2005-2010. The OR is represented by the pink line and the 95% CI is represented by the shaded part.

The non-linear relationship between HOMA-IR and CrD was further investigated. After adjusting for all confounding factors, the results demonstrated that as HOMA-IR increased, the risk of CrD significantly increased (*P*-overall < .001). However, no significant non-linear relationship was observed between the two (*P*-non-linear = .087) (Figure 2).

### Subgroup Analysis

**Figure 3.**
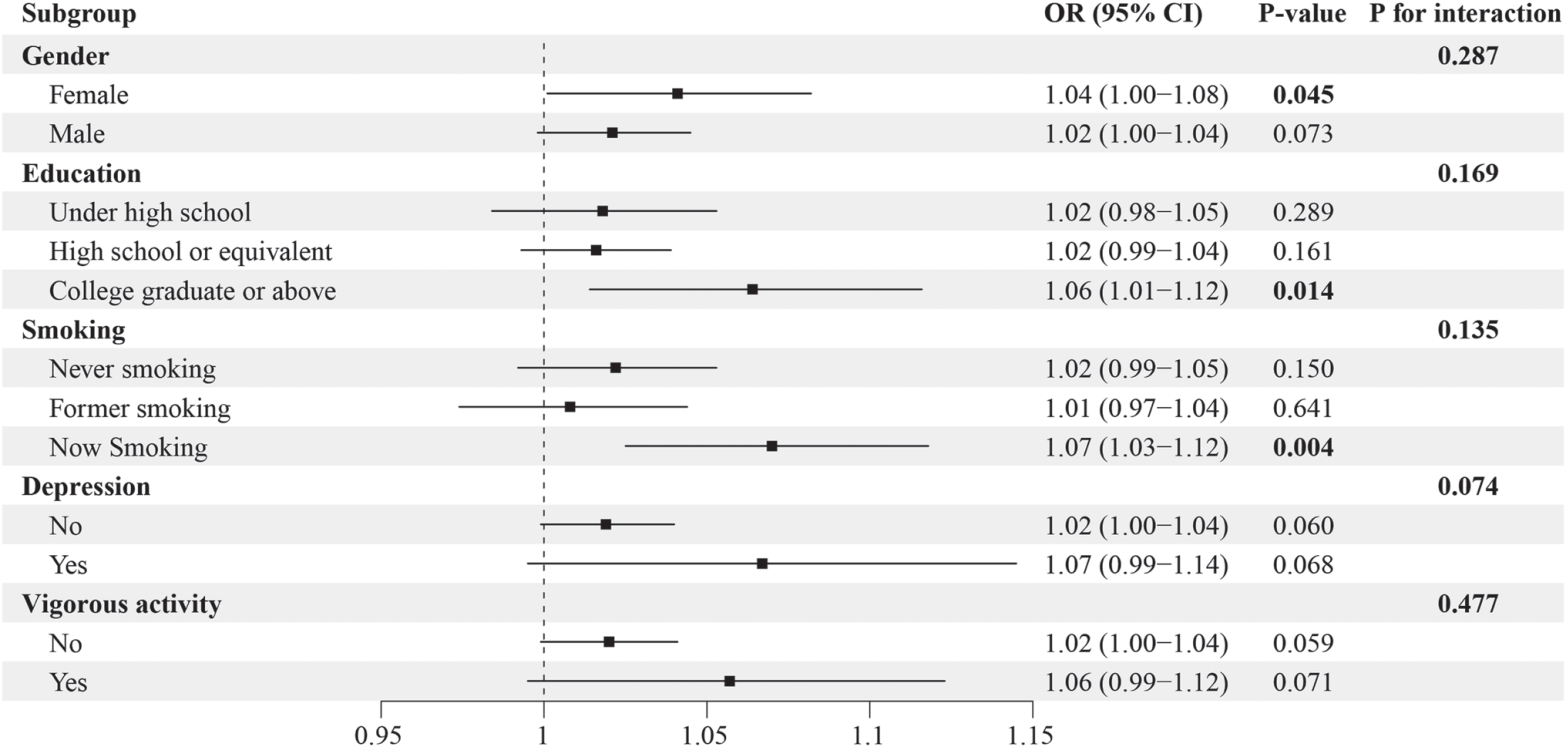
Subgroup analysis for the association between HOMA-IR and CrD, NHANES 2005-2010.

Next, subgroup analysis was conducted to explore the heterogeneity of the association between HOMA-IR and CrD in different characteristic populations. The positive linkage between HOMA-IR levels and CrD was only observed in females (OR = 1.04, 95% CI: 1.00-1.08, *P *= .045), people with a university and above education background (OR = 1.06, 95% CI: 1.01-1.12, *P *= .014), and current smokers (OR = 1.07, 95% CI: 1.03-1.12, *P *= .004), while no significant link between the 2 was found in other subgroups. Notably, despite the effect differences between the subgroups mentioned above, the interaction analyses revealed that gender, educational background, smoking status, depression, and intense physical activity did not interact with HOMA-IR (Figure 3).

### Mediation Analysis

**Figure 4.**
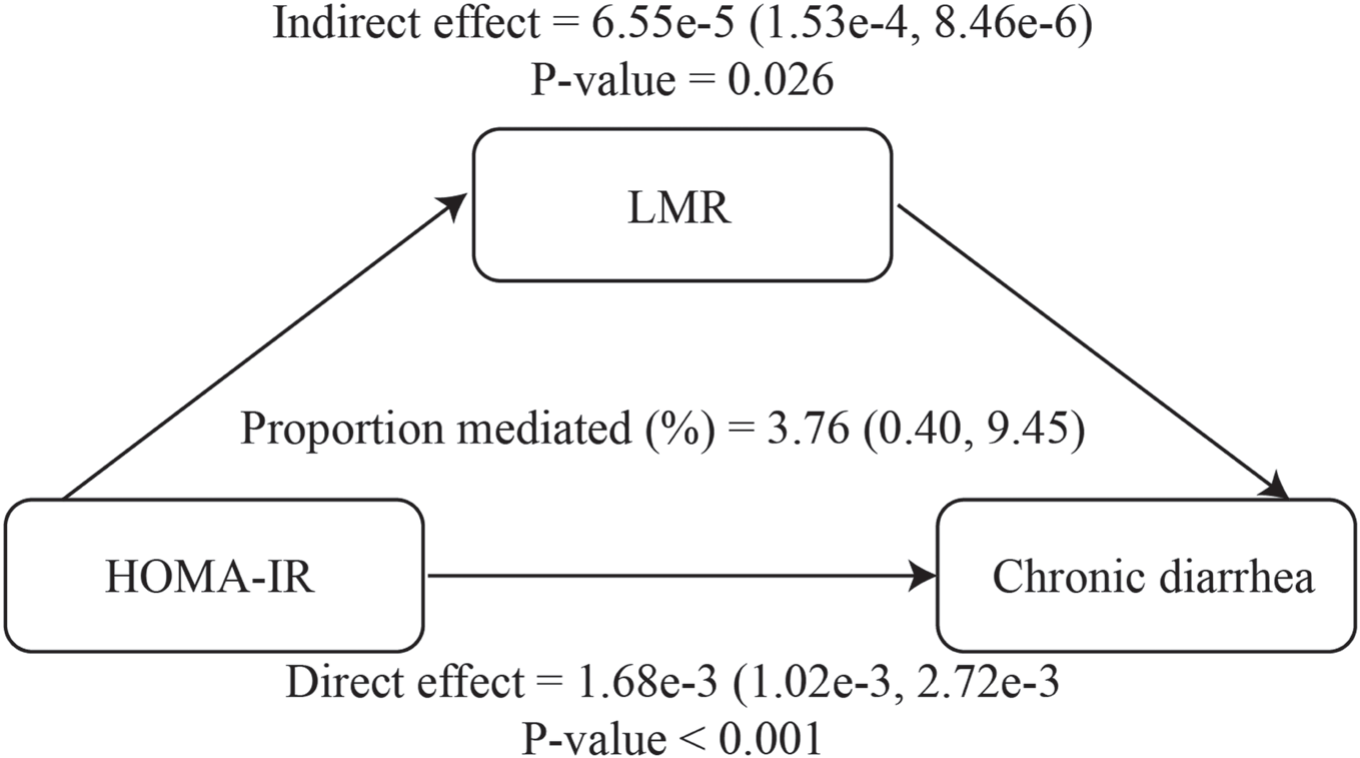
LMR functions as a mediator in the association between HOMA-IR and CrD, NHANES 2005-2010.

The mediating role of LMR in the association between HOMA-IR and CrD was further analyzed. The LMR partially mediates the association between HOMA-IR and CrD, with a mediation ratio of 3.76% (Figure 4).

## Discussion

This study, based on large sample data from the NHANES database, systematically evaluated the linkage between HOMA-IR and CrD, as well as the mediating role of LMR. Elevated HOMA-IR levels were significantly linked with the risk of CrD, and this association was more pronounced in women, highly educated populations, and current smokers. Furthermore, LMR played a partial mediating role between HOMA-IR and CrD.

The HOMA-IR, as a simple assessment method of IR, has been widely used to predict diabetes, hypertension, and cardiovascular adverse events.^18^ This study demonstrated that compared to the first quartile array of HOMA-IR, the fourth quartile array had a 68% elevated risk of CrD. The result is consistent with the positive correlation between the TyG index and CrD in previous studies, which collectively supports the linkage between IR and CrD.^6^ In addition, the improved TyG index, including TyG-waist circumference, TyG-waist to height ratio, and TyG-body mass index, combined with indicators reflecting obesity, is considered to better reflect IR and correlate closely to various digestive system diseases.^19^ It is worth noting that the prevalence of CrD in diabetes patients is significantly higher than that in non-diabetes patients (11.2% vs. 6.0%). This epidemiological evidence further emphasizes the role of IR in CrD.^5^ In addition, in the baseline data of this study, the proportion of patients with CrD suffering from depression was higher than that of non-CrD patients (14% vs. 5.7%, *P *< .001). This finding is consistent with previous studies, where an NHANES-based study also found that patients with depression had a higher incidence of CrD (15.53% vs. 6.05%).^20^ Depression may affect gut health through dysfunction of the brain-gut axis and abnormal gastrointestinal motility, thereby increasing the risk of CrD.^21^^-^^23^

The IR may amplify the risk of CrD through multiple pathways. First, diabetes and IR may lead to severe peripheral, autonomic, and central neuropathy. Abnormal insular activity is associated with gastrointestinal symptoms related to diabetes, suggesting that neuromodulation disorders may play a role in the occurrence of CrD.^24^ Secondly, intestinal peristalsis in patients with diabetes is slowed down,^25^ accompanied by dysbiosis of gut microbiota.^26^ A study based on the gut microbiome’s metagenomic profile found that in individuals with type 2 diabetes, the abundance of probiotic bacteria involved in infection prevention decreased, while multiple opportunistic pathogens increased.^27^^,^^28^ This may interfere with the normal functioning of the gut, activate inflammation, and elevate infection risk, thereby leading to diarrhea. In addition, IR may also affect intestinal function through disturbances in bile acid metabolism. In individuals with obesity and IR, studies have observed a great increase in markers of bile acid synthesis.^29^ Under normal circumstances, insulin infusion can rapidly reduce serum bile acid levels in non-obese subjects, but this regulatory effect is substantially weakened in obese individuals.^29^ Excessive bile acid entering the colon can affect electrolyte balance, leading to a decrease in water absorption by the colonic mucosa and accelerated peristalsis, thereby resulting in diarrhea.^30^ However, the molecular mechanisms by which IR specifically affects CrD are still not fully understood. Future research should further probe into the role of insulin signaling pathways in maintaining gut barrier homeostasis, neuroendocrine regulation, and inflammation-immune responses. In summary, the findings underscore the potential role of IR in the occurrence of CrD, further supporting the broad impact of metabolic disruptions on gastrointestinal health.

Subgroup analysis demonstrated that the association between HOMA-IR and CrD was more significant in females, current smokers and people with a university and above education background. A previous study investigated the relationship between obese adult patients in the United States and the incidence of CrD, showing that the incidence of CrD in obese women was significantly higher than that in obese men (10.24% vs. 5.95%, *P *< .001).^31^ Irritable bowel syndrome, as a possible cause of CrD, is also more prevalent in women, and this dramatic gender difference may be related to sex hormones.^32^^,^^33^ Regarding smoking status, smoking has been identified as a clear risk factor for microscopic colitis.^34^ This finding may partially explain why IR has a more prominent impact on gut health in this population. It is worth noting that the pronounced association observed at a higher education level is a discovery worthy of further exploration. This finding may be related to the specific dietary patterns in this population, such as more consumption of high fiber, high FODMAP foods, or artificial sweeteners in pursuit of health, which can induce diarrhea in susceptible individuals.^35^^,^^36^ Lifestyle factors, such as higher levels of work pressure affecting gut function through the brain-gut axis, may also affect the association between HOMA-IR and CrD.^37^^,38^ People with a university and above education background may have stronger health awareness, which may also lead to a higher reporting rate of intestinal symptoms. Women, current smokers and people with a university and above education background may need to pay closer attention to their IR, potentially by targeted measures such as minimizing high-glycemic and high-fat food intake, engaging in regular exercise, quitting smoking or reducing tobacco exposure, and managing psychological stress, all of which can lower the risk of CrD.^39^

The mediation analysis of this study suggested that LMR partially mediates the linkage between HOMA-IR and CrD. The LMR reflects the inflammatory state and immune balance of the body, and lower LMR is linked with higher disease activity in ulcerative colitis and rheumatoid arthritis.^8^^,^^40^ According to a previous study, IR promotes monocyte recruitment and induces polarization into pro-inflammatory M1 macrophages, leading to inflammation.^41^ Inflammation can impair gut barrier function and increase intestinal permeability, thus reinforcing diarrhea.^42^ Nevertheless, in this study, the mediating ratio of LMR between HOMA-IR and CrD was only 3.76%, indicating that inflammation may only constitute part of the mechanism, and future studies should delve further into the multifaceted impacts of IR on gut health.

This study has certain limitations. First, as a cross-sectional study, it cannot infer the causal relationship between HOMA-IR and CrD. Second, the lack of gut-specific indicators (such as gut microbiota and permeability data) and other inflammatory markers (such as neutrophil/lymphocyte ratio) in the NHANES database limits the further exploration of potential mechanisms. In addition, although multiple covariates were adjusted for as much as possible, confounding factors that are not systematically included—such as infection status and drug use (such as antibiotics, metformin, etc.)—could not be fully controlled, which may lead to residual confounding. It is worth noting that patients in the CrD group are older, which further suggests that age-related polypharmacy and comorbidity burden may be confounding factors that have not been fully controlled. Nevertheless, this study still provides valuable preliminary evidence for the association between IR and CrD, and future prospective studies are needed to include more comprehensive clinical and biological variables to validate the relationship between the two.

In conclusion, elevated HOMA-IR levels are significantly associated with the risk of CrD, and this association is more pronounced in women, those with higher education levels, and current smokers. Lymphocyte-to-monocyte ratio plays a partial mediating role in the linkage between HOMA-IR and CrD. This discovery reveals the potential role of IR in the occurrence of CrD, emphasizing the importance of preventing and managing CrD from the perspectives of metabolic regulation and inflammatory intervention.

## Supplementary Materials

Supplementary Material

## Figures and Tables

**Table 1.** Characteristics of NHANES Participants Between 2005 and 2010

	**Overall (N = 4984)**	**Non-chronic Diarrhea (N = 4612, 93.5%)**	**Chronic Diarrhea (N = 372, 6.5%)**	***P***
Age (years)	46.99 (16.28)	46.85 (16.35)	48.96 (15.02)	.048
Gender				.263
Female	2458 (50.0)	2259 (50.0)	199 (54.0)	
Male	2526 (50.0)	2353 (50.0)	173 (46.0)	
Race				.326
Mexican American	884 (7.6)	801 (7.4)	83 (9.9)	
Other Hispanic	408 (3.9)	371 (3.9)	37 (4.9)	
Non-Hispanic White	2648 (74.0)	2476 (74.0)	172 (70.0)	
Non-Hispanic Black	846 (9.3)	779 (9.2)	67 (10.0)	
Other race	198 (5.3)	185 (5.3)	13 (4.9)	
Education				<.001
Under high school	511 (5.3)	434 (4.8)	77 (12.0)	
High school or equivalent	1976 (36.0)	1822 (36.0)	154 (39.0)	
College graduate or above	2497 (59.0)	2356 (59.0)	141 (49.0)	
Marital status				.479
Never married	742 (16.0)	693 (16.0)	49 (13.0)	
Married/living with partner	3146 (67.0)	2908 (67.0)	238 (67.0)	
Widowed/divorced/separated	1096 (18.0)	1011 (17.0)	85 (20.0)	
PIR				.060
≤1.3	1380 (17.0)	1244 (17.0)	136 (23.0)	
1.3-3.5	1943 (37.0)	1804 (37.0)	139 (39.0)	
>3.5	1661 (46.0)	1564 (46.0)	97 (38.0)	
Smoking				.024
Never smoking	2574 (52.0)	2398 (52.0)	176 (45.0)	
Former smoking	1353 (26.0)	1253 (27.0)	100 (26.0)	
Current Smoking	1057 (22.0)	961 (21.0)	96 (29.0)	
Alcohol drinking				.807
No	1349 (23.0)	1241 (22.0)	108 (23.0)	
Yes	3635 (77.0)	3371 (78.0)	264 (77.0)	
Depression				<.001
No	4600 (94.0)	4291 (94.0)	309 (86.0)	
Yes	384 (6.2)	321 (5.7)	63 (14.0)	
Vigorous activity				.364
No	3262 (60.0)	3002 (60.0)	260 (63.0)	
Yes	1722 (40.0)	1610 (40.0)	112 (37.0)	
ABSI	137.53 (11.95)	137.57 (11.89)	137.05 (12.72)	.532
Total sugar intake (gm)	115.92 (66.98)	115.65 (66.95)	119.83 (67.37)	.324
Dietary fiber intake (gm)	16.50 (8.39)	16.48 (8.16)	16.79 (11.28)	.750
Total fat intake (gm)	82.09 (39.75)	81.98 (39.65)	83.74 (41.23)	.630
Caffeine intake (mg)	186.81 (189.22)	185.59 (187.99)	204.32 (205.49)	.179
Moisture intake (gm)	2938.12 (1197.20)	2931.17 (1188.32)	3037.76 (1315.82)	.388
LMR	4.03 (1.55)	4.02 (1.53)	4.22 (1.70)	.053
HOMA-IR	3.24 (3.83)	3.19 (3.78)	3.99 (4.39)	.003
HOMA-IR quartiles				.055
Q1 (≤1.37)	1042 (25.0)	990 (25.0)	52 (19.0)	
Q2 (1.37-2.25)	1209 (25.0)	1133 (25.0)	76 (23.0)	
Q3 (2.25-3.92)	1294 (25.0)	1191 (25.0)	103 (26.0)	
Q4 (>3.92)	1439 (25.0)	1298 (24.0)	141 (32.0)	

ABSI, A Body Shape Index; HOMA-IR, homeostatic model assessment of insulin resistance; LMR, lymphocyte-to-monocyte ratio; PIR, poverty income ratio.

**Table 2.** The Odds Ratios Between HOMA-IR and CrD, NHANES 2005-2010

	**Crude**		**Model I**		**Model II**	
	**OR (95% CI)**	***P***	**OR (95% CI)**	P	**OR (95% CI)**	P
HOMA-IR	1.03 (1.01, 1.06)	.003	1.03 (1.01, 1.05)	.009	1.03 (1.00, 1.05)	.022
HOMA-IR quartiles						
Q1 (≤ 1.37)	Ref		Ref		Ref	
Q2 (1.37-2.25)	1.21 (0.75, 1.97)	.426	1.16 (0.71, 1.89)	.534	1.23 (0.74, 2.05)	.411
Q3 (2.25-3.92)	1.41 (0.84, 2.37)	.190	1.34 (0.79, 2.28)	.275	1.41 (0.81, 2.45)	.210
Q4 (> 3.92)	1.75 (1.14, 2.69)	.012	1.61 (1.04, 2.49)	.033	1.68 (1.03, 2.74)	.040

No covariates were adjusted in the crude model. Model I was adjusted for age, gender, race, and educational level. Model II was adjusted for age, gender, race, educational level, marital status, PIR, ABSI, smoking status, alcohol drinking, depression, vigorous activity, total sugars intake, dietary fiber intake, total fat intake, caffeine intake, moisture intake, and LMR.

**Table:** 

Code or Value	Value Description
1	Type 1 (separate hard lumps, like nuts)
2	Type 2 (sausage-like, but lumpy)
3	Type 3 (like a sausage but with cracks in the surface)
4	Type 4 (like a sausage or snake, smooth and soft)
5	Type 5 (soft blobs with clear-cut edges)
6	Type 6 (fluffy pieces with ragged edges, a mushy stool)
7	Type 7 (watery, no solid pieces)
77	Refused
99	Don’t know
