# Supplementary material for: Deciphering the Association Between Homeostatic Model Assessment of Insulin Resistance and Chronic Diarrhea and the Mediating Role of Lymphocyte-to-Monocyte Ratio
Source: Turk J Gastroenterol. 2026 Jan 26;37(5):555–63. doi: 10.5152/tjg.2026.25247 (PMC13182916; doi:10.5152/tjg.2026.25247)
Supplement: Supplementary Material [file supplementary_material.pdf]

SUPPLEMENTARY MATERIAL. QUESTIONNAIRE

The Bowel Health section of the MEC interview provides personal interview data on fecal incontinence and defecating function for adults age 20 year and older.

Question BHQ060 is the Bristol Stool Form Scale. The stool consistency scale has been used in a series of studies. These studies have shown that a subject's rating of his/her own individual radiopaque markers to pass through the gastrointestinal tract.

BHQ060-Common Stool Type

English Text: Please look at this card and tell me the number that corresponds to your usual or most common stool type.

Code or Value	Value Description
1	Type 1 (separate hard lumps, like nuts)
2	Type 2 (sausage-like, but lumpy)
3	Type 3 (like a sausage but with cracks in the surface)
4	Type 4 (like a sausage or snake, smooth and soft)
5	Type 5 (soft blobs with clear-cut edges)
6	Type 6 (fluffy pieces with ragged edges, a mushy stool)
7	Type 7 (watery, no solid pieces)
77	Refused
99	Don't know
